# A Case of Poisoning with *Abrus precatarius* Complicated with Bilateral Basal Ganglia Haemorrhage

**DOI:** 10.1155/2022/3318197

**Published:** 2022-08-25

**Authors:** Arun Rajaratnam, Kishan Dissanayake, Kiertie Kularatne, Sunil Bowattage

**Affiliations:** Teaching Hospital Kandy, Kandy, Sri Lanka

## Abstract

*Abrus precatarius* is a tropical climber, whose seeds contain abrin, which is known to cause toxicity in humans. We report a case of a young girl, who presented with haemorrhagic enterocolitis, bilateral septal vein thrombosis, and basal ganglia haemorrhage leading to seizures and coma, following ingestion of toxic *A. precatarius* seeds. To the best of our knowledge, this is the first ever case to describe such an intracranial complication of abrin poisoning.

## 1. Introduction

*Abrus precatarius*, also referred to as rosary pea or jequirity pea, is a woody, Leguminosae climber found in many parts of Asia and Australia. The plant is widely distributed in Sri Lanka and its seeds are used in local board games and are used in Ayurvedic medical practice. The seeds are small, ovoid, glossy, and scarlet coloured with a black eye at the hilum, making those attractive for accidental consumption by children. Despite this, cases of abrin poisoning are rarely encountered and reported from the country. The seeds, roots, and leaves are considered poisonous, but toxicity occurs commonly from accidental or deliberate consumption of seeds. The hard protective outer covering needs to be breeched for the toxic contents to be released into the alimentary tract. Seeds of *A. precatarius* are toxic primarily due to a potent haemagglutinin called *Abrus agglutinin* and four other water-soluble toxic abrin (named “*a*” to “*d*”) that are eliminated via the renal route [1]. Abrin (lectins) contains two subunits (A and B chains) involved in toxic pathogenesis. The B chain facilitates the intracellular entry of the A chain by binding to galactosyl terminated cell surface receptors, whereas the A chain inactivates 60 S ribosomal activity of Elongation Factors (1 and 2) leading to impaired protein translation and eventual cell death [2]. The abrin content of the seeds is about 0.15% w/w and a dose as small as 0.1–1 *μ*g/kg body weight is said to be fatal [3]. Common toxic manifestations develop after a latent period of around 3 days [3] and include nausea, vomiting, diarrhoea, gastrointestinal bleeding, focal necrosis of the kidneys and liver, haemagglutination, haemolysis, hypovolemic shock, retinal haemorrhages, central nervous system complications, and death even after 14 days. Symptoms may persist for even more than 10 days [4]. The mortality rate is usually around 5% [5]. The case we are reporting is an intracranial complication of basal ganglia haemorrhage, which was not previously described in association with *A. precatarius.*

## 2. Case Presentation

**Figure 1:**
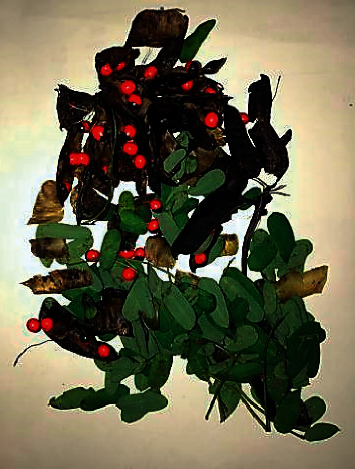
*A. precatarius* pod with seeds.

**Figure 2:**
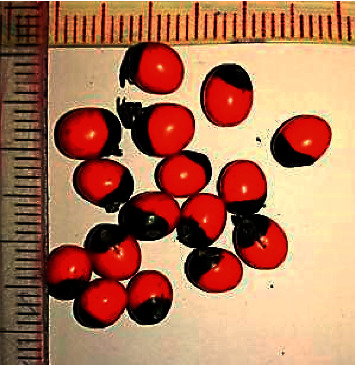
*A.precatarius* seeds, from a plant in the patient's neighbourhood.

A previously well, 17-year-old, unmarried, Sinhalese school girl presented after deliberate consumption of four seeds of *A. precatarius* plant from her garden (Figures 1 and 2). Following a family dispute, she had chewed and swallowed those unaware of the fatal outcome. The following day, she had developed painful lower abdominal cramps with small-volume watery diarrhoea. The day after, she had mild chills without much diarrhoea. On the fourth day, abdominal cramps and small-volume mucoid to watery diarrhoea had returned and persisted, making her seek medical attention. She had no fever or vomiting. She had no previous or family history of thrombotic conditions, nor was she on any medications such as oral contraceptive pills (OCPs). Her last regular menstruation was around a week prior to admission.

At admission, she was thin-built, had a body mass index (BMI) of 19 kg/m^2^, was conscious, was rational, and had stable vitals (core temperature of 37°C, pulse rate 110/minute, blood pressure-102/86 mmHg, respiratory rate of 29 breaths/minute and on-air arterial oxygen saturation (Sp_a_O_2_) of 99%. The rest of the examination was unremarkable apart from mild pallor, voluntary guarding, and tenderness of the lower abdomen. Rectal examination did not reveal any blood. She was initially treated with opiate analgesics, hydrated with intravenous normal saline and oral rehydration solution, and empirical antibiotics (intravenous meropenem and intravenous metronidazole), to cover possible infective gastroenteritis.

On the fifth day morning, she complained of fresh per rectal bleeding mixed with stools, had a slight drop in blood pressure, and required a transfer to the intensive care unit for monitoring. Later that night, she also had spontaneous vaginal bleeding. Urine human chorionic gonadotropin (hCG) was negative and serum beta hCG was low (< 2.39 mIU/ml). An abdominopelvic ultrasound revealed a normal liver, normal kidney size (measuring 9 cm bilaterally) and echotexture, a small amount of free fluid in the pelvic cavity, a slightly bulky and cystic right ovary, and an endometrial thickness of 0.8 cm without any evidence of intrauterine or extrauterine pregnancy. She was given a single dose of intravenous tranexamic acid (500 mg) to reduce the bleed. A few minutes later, she developed a generalised tonic-clonic seizure lasting for more than ten minutes with abnormal flexing, fixing of gaze, stridor, and subsequent drop in the Glasgow Coma Scale (GCS) from 15 to 10 and Sp_a_O_2_ to 70%, warranting immediate intubation and ventilation. Her hydration, blood pressure, and oxygenation status were normal at the time of the seizure. There was no papilloedema, neck stiffness, or focal neurological signs. Apart from a mild hyponatremia, the rest of the metabolic parameters including blood sugar were normal. A noncontrast computed tomogram (NCCT) of the brain performed within a few hours of intubation did not reveal any haemorrhage or cerebral oedema. An urgent MRI could not be performed at that moment. Following a specialist neurology consultation, she was commenced on a five-day course of intravenous methylprednisolone, suspecting of *A. precatarius* induced demyelination, along with intravenous antiepileptics (midazolam, thiopentone sodium, phenytoin, and levetiracetam) and oral topiramate for refractory seizures.

Serial neurological examinations were conducted daily, while under sedation and being ventilated did not reveal any signs of focal neurology, papilloedema, pupillary abnormalities, or signs of meningism. However, she was noticed to have generalised flaccidity with intermittent, brief (about 10 seconds) episodes of tonicity and involuntary movements of the extremities, hyporeflexia, and episodes of involuntary blinking. Digital electroencephalogram (EEG) performed while ventilated but off sedation, revealed continuous polymorphic delta activity that is suggestive of moderate generalised cerebral dysfunction. Epileptiform discharges were not seen in the EEG. The patient's parents did not consent for a lumbar puncture.  Table 1 gives the trend of biochemical investigations.  Table 2 gives the trend in arterial blood gas parameters. Serial blood pictures were revealing evidence of inflammation and iron deficiency with recent bleeding without haemolysis. Stool for occult blood was positive even during the second week. Her thryoid stimulating hormone (TSH) was 0.659 mIU/L·0(0.4–4.0), and free thyroxine (T4) was 8.50 ng/dL (10.3–25.3). She had a negative antinuclear antibody (ANA), which was found out after undergoing the Coombss test and PCR for SARS RNA. However, she had a high d-dimer (value: 1.2 mg/L, reference < 0.2 mg/L). Urine had no protein or active sediment. Serial blood, urine, and endotracheal cultures were negative. Active limb physiotherapy was offered. Measures were taken to prevent pressure sores, aspiration, and venous thromboembolism (pneumatic compression stockings and low molecular weight heparin). With no marked improvement in neurology following a five-day course of methylprednisolone, she was commenced on seven daily cycles of plasma exchange cycles (day 12 to day 17 of illness) with FFP cover, replacing 1800 ml of plasma in each session. Although her diarrhoea settled, she had stool occult blood positivity. She was extubated and sedatives were withheld completely on the 14^th^ day of illness, following which she initially was able only to turn her head sideways and to turn her eyes laterally, spontaneously, and as well as on command. Neurological examination revealed flaccid limbs with proximal and distal weakness (grade 3/5), hyporeflexia, down-going plantar response, cognitive dysfunction manifested by amnesia and poor attention, drowsiness, and difficult phonation.

**Figure 3:**
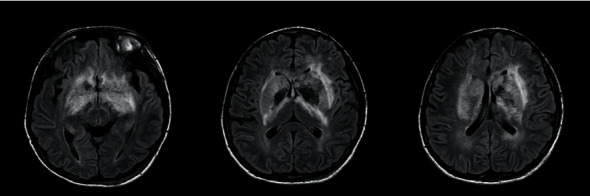
T2-FLAIR image, an axial section at basal ganglia.

**Figure 4:**
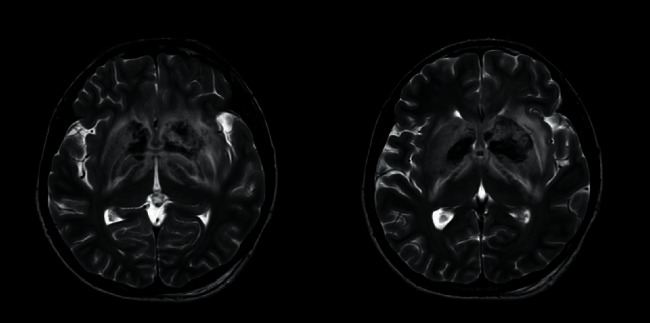
T2-W image, an axial section at basal ganglia.

**Figure 5:**
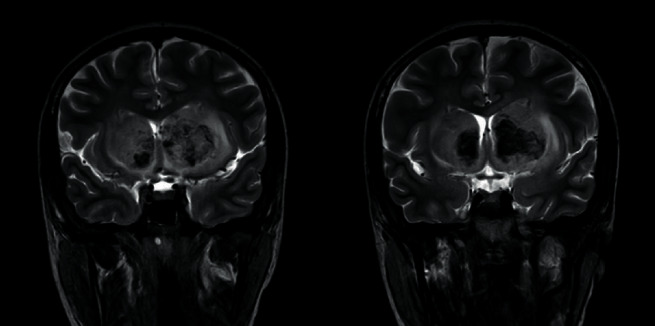
T2-W image, a coronal section at basal ganglia.

**Figure 6:**
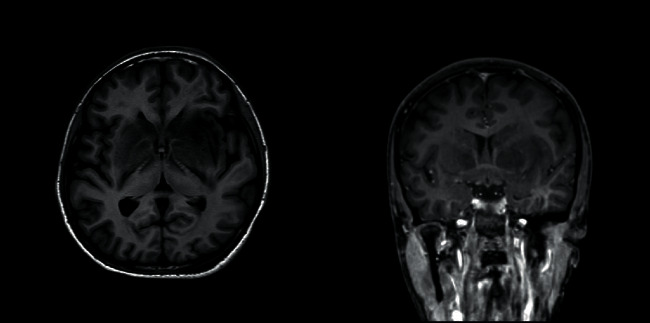
T1-W, an axial section and T1-W gadolinium contrast, a coronal section images at basal ganglia.

**Figure 7:**
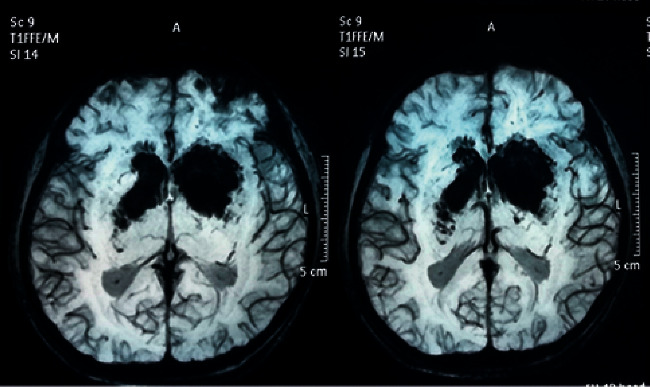
SWI image, an axial section at basal ganglia.

**Figure 8:**
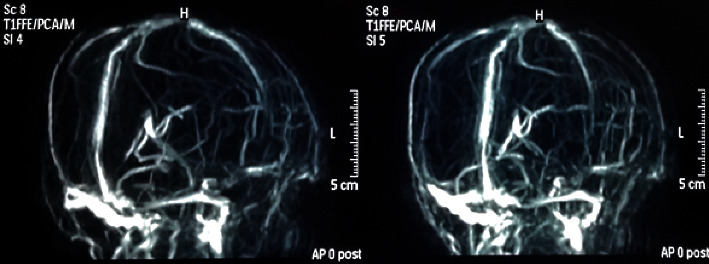
CT venogram.

Magnetic resonance imaging (with T1W, T2W, FLAIR, DWI, GAD, MRV, and MRA sequences) performed on the 15th day of poisoning showed bilateral, symmetric, ill-defined, heterogenous T2 weighted (T2W), and fluid attenuated inversion recovery (FLAIR) showed heterogenous (high with patchy central low) signal abnormality in striato-capsular areas of basal ganglia with thalami sparing (Figures 3–5). The low-intensity areas seen in T2W and FLAIR sequences showed intermediate signal intensity in T1 weighted (T1W) and blooming on susceptibility weighted imaging (SWI), with no enhancement of contrast, suggesting evidence for bilateral, symmetrical haemorrhage with associated vasogenic oedema involving basal ganglia and internal capsule (Figures 6 and 7). Cerebral sulci, gyri, subcortical white matter, ventricular system, brainstem, and cerebellum were normal with preserved grey and white differentiation. Bilateral septal veins visualized on SWI are not opacified in contrast to magnetic resonance venography (MRV) favouring bilateral septal vein thrombosis (Figure 8). Bilateral internal cerebral veins and thalomostriate veins, great vein of Galen, and straight sinus were normal. The rest of the MRV did not show major dural venous sinus thrombosis. Magnetic resonance arteriography (MRA) was normal. Interval MRI in 1 week showed persistence of previously noted basal ganglia haemorrhage with no new intracerebral haemorrhage.

On day 18, she was in a normal ward, where physiotherapy, speech therapy, neurorehabilitation, nasogastric feeding, levetiracetam, and intravenous antibiotics were continued. Although she found difficulty in phonation and had impaired bladder sensation, there was a gradual improvement in comprehension, cognitive function, and limb power. She was discharged on the 24^th^ day with oral levetiracetam and haemetinics and was emphasised on home-based physiotherapy and NG feeding. Modified Rankin Scale (MRS) was 4 on discharge. Oral feeding was commenced and the urinary catheter was removed 1-week postdischarge.

During a review at 1 month postdischarge, she was found to be alert, verbalising, scored 27/30 on the validated Sinhalese version Montreal Cognitive Assessment (MoCA) [6], had improving limb functions except for a left-sided foot drop, and had an MRS of 1. Nerve Conduction Studies of the left lower limb only revealed evidence of a moderate partial common peroneal lesion at the fibular neck with active denervation and no reinnervation. She had lost weight (BMI 13.7 kg/m^2^; midarm circumference of 17.5 cm), had minor nutritional insufficiencies (haemoglobin 9.6 g/dL, mean corpuscular volume 91 fL, vitamin D level 28.4 *n*g/ml, vitamin B12 level 262.2 pg/mL, serum ferritin 169.0 mg/mL, and serum iron 6.73 *μ*mol/L), and had a patch of nonscarring alopecia. Antinuclear antibody level was nonsignificant (titre of 1 : 40). She had completely recovered functionally and cognitively at 2 months postdischarge. Nutritional supplementation was continued and levetiracetam was gradually tapered and omitted after 6 months but had to be recommenced at a low dose as seizures were recurring a few weeks after omission.

## 3. Discussion

Neurological consequences of abrin toxicity occur less frequently than gastrointestinal consequences. Reported neurological complications include headache, hallucinations, mydriasis, papilloedema, tetany, seizures, coma, and encephalitis-like syndrome [7, 8]. There are few cases of late-onset CNS immune-mediated demyelination, making us initially suspect it as the underlying pathological process in our patient [9–11]. CNS demyelination is due to abrin's immunomodulatory and immunostimulatory properties [12, 13]. Abrin-induced demyelination is reported to respond completely to methylprednisolone and plasma exchange [9–11]. A case of osmotic demyelination due to direct effects of abrin in the absence of serum sodium abnormalities was reported previously in a 2-year-old [14]. Thrombotic complications with abrin are sparsely reported. One case of superior sagittal sinus thrombosis attributed to dehydration from gastrointestinal loss, capillary leak syndrome, or from direct endothelial injury from abrin has been reported [15]. Abrin agglutinator causes haemagglutination. However, the exact effect of abrin on coagulation is unknown. Our patient had a normal coagulation profile despite bleeding and thrombosis occurring simultaneously.

MRI and MRV are the most sensitive tools in detecting early deep cerebral vein thrombosis (CVT) [16]. The difference in signal intensities of T1 and T2 weighted images may help to date the onset of thrombosis and subsequent vasogenic oedema and ischaemia [17]. Isolated thrombosis of bilateral septal veins is not frequently described. The bilateral septal veins, which drain the anterior basal ganglia, corpus callosum, and the deep medullary frontal white matter, begin at the lateral aspect of the anterior horns of lateral ventricles and then pass medially and inferior to the genu of the corpus callosum. These then turn posteriorly, traverse the septum pellucidum, and join with the thalamostiate veins behind the foramen of Monro to form the internal cerebral vein [18].

Abrin per se is described to induce endothelial injury leading to increased capillary permeability and vasogenic oedema [2, 19]. In this reported case, abrin's direct inhibitory effect on protein synthesis of the metabolically active basal ganglia may have resulted in tissue inflammation, ischaemic necrosis, and subsequent bleeding. Thrombosis of the thin calibre septal veins that traverse adjacent to the anterior basal ganglia may have occurred secondary to surrounding inflammation. Another possibility is that the direct endothelial injury by abrin activates the clotting cascade leading to bilateral septal vein thrombosis first, thereby leading to vasogenic and cytotoxic oedema, and later haemorrhage of the draining basal ganglia. The metabolically active basal ganglia are commonly targeted in other poisonings as well. Bilateral basal ganglia haemorrhages had been previously described due to carbon monoxide, methanol, ethylene glycol, and following scorpion sting [20–23]. Therefore, in the context of poisoning, MRI changes in our patient are likely to represent basal ganglia swelling which has subsequently undergone haemorrhagic transformation due to necrosis. The spatial resolution of MRV is not enough to reliably comment on septal vein thrombosis. Poor visualisation of septal veins on MRV/SWI is likely to be secondary to the mass effect of the swollen basal ganglia, rather than septal vein thrombosis.

Our patient had no diagnostic MRI evidence for acute haemorrhagic encephalomyelitis (AHEM) and acute demyelinating encephalomyelitis (ADEM). Nevertheless, she was initially managed for possible immune-mediated demyelination with methylprednisolone and therapeutic plasma exchange, until an MRI diagnosis was available. Although a partial neurological recovery was noticed with plasma exchange, much of the neurological and cognitive recovery occurred on its own with the passage of time, perhaps also supported by active neuro-rehabilitating care during the initial months. Plasma exchange may have had an effect in removing both the toxin and immune modulators. Untreated patients with deep CVT often develop features of raised intracranial pressure and brain herniation, which can lead to permanent residual disability and often death. However, in our patient prophylactic enoxaparin, which was given during the initial period, had to be discontinued due to persistent faecal occult bleeding and dropping haemoglobin levels. Our patient had no absolute indication to be screened for hereditary or autoimmune thrombophilias.

Till date, there is no antidote for abrin poisoning. Hence, treatment for abrin poisoning is largely supportive and symptomatic with the provision of intravenous fluids, correction of electrolyte imbalance, and correction of anaemia, as in our case. Gastric lavage, activated charcoal, and induced emesis may be used in the early hours after ingestion, but need to be administered with caution due to the necrotizing action of abrin [24]. Alkaline diuresis with sodium bicarbonate will prevent the precipitation of haemoglobin and its products in kidney tubules [5]. Aggressive treatment and monitoring are warranted for all patients even though there is a delay in presentation.

It is important to note that our patient was brought for medical care with three days of symptoms of abrin poisoning, reflecting the public unawareness of the grave consequences of the poisoning. Had she not come at the correct time, a fatality may not have been prevented. Hence, it is pertinent to create awareness on the harmful effects of such poisonous plants in the tropics. In countries such as the United States, where *A. precatarius* is not commonly grown, a legislature on restricting the labelling and import of *Abrus* and *Abrus* derived items have been effective [1].

## 4. Conclusion

The reported case depicts a unique and unusual neurological consequence of abrin poisoning. It is not very clear why abrin would predispose to both cerebral venous thrombosis and haemorrhage. Nevertheless, one needs to consider venous thrombotic and haemorrhagic causes in addition to the well-described demyelinating causes, when managing abrin-related central neurological manifestations such as seizures and coma. Magnetic resonance imaging and additional sequences are effective in differentiating these. Further studies may be needed to understand the effects of abrin on coagulation.

## Figures and Tables

**Table 1:** Trend of blood or serum biochemical parameters.

Day	Reference range	5	6	7	8	10	12	14	18	20	24
WBC (X10^3^/*μ*L)	4.0–10.0	23.4	42.5	34.5	23.4	22.2	17.5	39.0	13.5	14.1	10.5
Neutrophil (X10^3^/*μ*L)	2.0–7.0	20.0	37.9	30.9	18.8	18.9	16.1	33.3	10.0	10.9	7.3
Lymphocyte (X10^3^/*μ*L)	0.8–4.0	1.4	0.7	1.3	1.5	1.2	0.8	2.7	1.9	1.9	2.2
Haemoglobin (g/L)	11.0–15.0	13.2	14.5	11.1	10.1	8.2	8.8	7.8	7.7	9.0	9.1
Haematoctit (%)	37.0–54.0	43.5	44.4	32.7	30.6	27.9	26.9	24.2	24.1	28.1	28.1
Platelet count (X 106/*μ*L)	150–40	161	123	110	140	181	202	287	349	313	268
ESR (mm/1^st^ hour)	< 15	5									30
Blood urea (mmol/L)	2.8–7.2	5.0	3.38	4.13	4.8	10.0	17.0	16.7	7.8	5.1	
Serum creatinine (*μ*mol/L)	65–120	49.0	63.6	75.8	64.3	92.8	107.0	142.7	87.8	57.4	53.7
Serum magnesium (mmol/L)	(0.7–1.0)				0.87	0.77	0.47	0.79	0.61		0.53
Serum phosphorus (mmol/L)	(0.8–1.45)		1.05		0.77	0.27	1.22	1.42	1.62		1.77
Serum total calcium (mmol/L)	(2.1–2.55)		1.8		1.83	1.80	1.86	1.87	2.17		2.22
Serum potassium (mmol/L)	3.5–5.5	4.0	3.5	3.9	4.7	4.1	4.2	3.8	4.0	4.2	4.0
Serum sodium (mmol/L)	1.5–150	131	130	128	132	135	138	139	144	143	140
Urine potassium (mmol/L)			14.1		15.7						
Urine sodium (mmol/L)			172	168.6	139.0						
Urine osmolality (mOsm/L)	300–900		683	565	877						
Serum osmolality (mOsm/L)	275–295	268		270	294						
ALT(U/L)	< 34	23.4	11.4	11.8	25.1	40.1	49.4	57.0	45.6	75.7	59.3
AST (U/L)	< 31	35.5	20.0	30.5	63.0	68.6	56.6	61.2	46.8	62.0	43.0
GGT (U/L)		10.8			11.7		23.1	31.0	33.9		67.9
ALP (U/L)		56.3						59.8	67.4		84.8
Total bilirubin (*μ*mol/L)	5.0–19.0	7.33			3.57	4.13	3.47	3.14	4.56		6.59
Direct bilirubin (*μ*mol/L)	1.7–6.8	2.19			1.31	1.47	0.62	0.71	1.57		2.32
Total protein (g/dL)	6.6–8.3	5.7	4.9	3.9	3.7	3.5	4.0	4.9	6.3		7.6
Serum albumin (g/dL)							2.4	2.8	3.7		4.2
APTT (sec)	32–35	32.8		30.2	36.2	28.9	22.1	35.0	33.3	32.2	36.7
PT/INR	0.9–1.1	1.13	1.42	1.22	1.31	1.21	1.09	1.30	1.02	1.05	1.16
LDH (U/L)	< 225	224									
CPK (U/L)	< 145				1184						164

**Table 2:** trend of arterial biochemical parameters.

Day	5	6	8	9	10	12	14	17
pH	7.33	7.31	7.35	7.44	7.56	7.43	7.48	7.45
pCO2	27.8	32.5	34.8	25.2	29.2	34.5	30.2	36.3
pO2	60.4	69.0	84.9	133.9	198	52	123.6	82.4
HCO3−	14.5	16.5	19.2	17.3	21.0	22.9	22.5	27.1
Lactate	1.0	2.5	1.4	1.2	1.5	1.6	1.3	0.4

## Data Availability

The data are available from the corresponding author upon request.
